# Manganese dioxide nanozyme alleviates PEDV GIIc-associated growth retardation in neonatal piglets by improving antioxidant status, intestinal morphology, and manganese homeostasis

**DOI:** 10.3389/fvets.2026.1963407

**Published:** 2026-10-06

**Authors:** Jintao Wang, Xin Lai, Mingfang Hu, Sheng Liang, Tianci Wu, Xiyin Fan, Yuheng Luo, Jun He, Lianqiang Che, Daiwen Chen, Aimin Wu

**Affiliations:** 1 Institute of Animal Nutrition, Sichuan Agricultural University, Chengdu, China; 2 Key Laboratory for Animal Disease-Resistance Nutrition of China Ministry of Education, Sichuan Agricultural University, Chengdu, China

**Keywords:** antioxidant capacity, growth performance, gut microbiota, intestinal morphology, manganese dioxide nanozyme, manganese metabolism, neonatal piglets, porcine epidemic diarrhea virus

## Abstract

**Introduction:**

Porcine epidemic diarrhea virus (PEDV) infection threatens swine production, and growth retardation in neonatal piglets born to infected sows requires practical intervention. This study evaluated the effects of oral manganese dioxide (MnO_2_) nanozyme supplementation on growth and physiological indices in these piglets.

**Methods:**

In this study, 16 litters from PEDV GIIc-infected sows were assigned to four treatment groups according to body condition and PEDV RT-qPCR Ct value, with four litters from uninfected sows used as the negative control (NC). From 3 d of age to 21 d of age, piglets from PEDV-infected sows received oral MnO_2_ nanozyme at different concentrations every other day.

**Results:**

Compared with NC group, PEDV-positive control (PC) group had lower average daily gain and jejunal villus height (*P* < 0.05), decreased serum CAT activity and serum GSH concentration, reduced jejunal GSH concentration, altered manganese transporter expression in the duodenum, and increased hepatic manganese accumulation. In piglets, oral MnO_2_ nanozyme restored average daily gain to normal levels, improved jejunal villus height, and maintained GSH concentrations in serum, jejunum, and liver at levels not different from NC. MnO_2_ nanozyme administration also increased duodenal DMT1 expression, decreased hepatic expression of transporters such as ZIP8, tended to increase duodenal manganese content, reduced hepatic manganese-transporter expression relative to the PC group, and alleviated hepatic manganese accumulation. In rectal fecal samples from piglets, MnO_2_ nanozyme increased the relative abundance of beneficial bacterial genera, including *Prevotella* and *Bifidobacterium*.

**Discussion:**

Oral MnO_2_ nanozyme supplementation was associated with alleviation of PEDV GIIc-associated growth retardation and changes in intestinal morphology, antioxidant status, and manganese-related indices. The 5 mg/L group showed a favorable growth response at the lowest tested concentration. Baseline body-weight differences should be considered when interpreting these findings, and further controlled studies are needed to establish the underlying mechanisms and optimal administration regimen.

## Introduction

Porcine epidemic diarrhea (PED) is a highly contagious intestinal disease caused by the porcine epidemic diarrhea virus (PEDV). Characterized by vomiting, watery diarrhea, and high mortality rates in suckling piglets, it has inflicted substantial economic losses on the global swine industry (1, 2). In recent years, PEDV variants have spread widely worldwide, particularly in China and neighboring Asian countries. Genotype II (GII) strains, characterized by insertion or deletion mutations at the S gene major antigenic site, have become the dominant circulating strains (3). Among these, the GIIc subtype, as one of the primary locally prevalent branches, exhibits rapid transmission and high pathogenicity, posing severe challenges to traditional vaccine immunization strategies (4).

In intensive commercial pig farms, “refeeding” or “acclimation” using disease materials derived from locally prevalent strains is a common production practice to rapidly establish herd immunity against specific circulating strains (5). This strategy aims to induce sows to produce high levels of secretory IgA (sIgA) specific to certain strains (e.g., PEDV GIIc), thereby providing passive immune protection to newborn piglets via colostrum (6). However, in actual production, the sow acclimation process is a double-edged sword. High-concentration viral exposure may not only prolong viral shedding in sows, leading to persistently elevated environmental viral loads, but also expose newborn piglets to the virus before their own immune systems are established (7). Studies indicate that even with some maternal antibody protection, early exposure to PEDV causes damage to intestinal epithelial cells in piglets, triggering severe inflammatory responses and oxidative stress (8).

Pregnant sows infected with PEDV not only show clinical signs such as abortion but also frequently give birth to neonatal piglets that suffer from growth retardation (9, 10). Oxidative stress is recognized as a key mechanism underlying viral-induced intestinal barrier dysfunction and growth retardation (11). The antioxidant system in newborn piglets is immature, making it difficult to effectively clear excess reactive oxygen species (ROS) induced by viral infection. This redox imbalance further exacerbates intestinal villus atrophy, reduces nutrient absorption efficiency, and identifying a safe, efficient formulation capable of specifically scavenging excess ROS in the intestine is crucial for mitigating the adverse effects of PEDV GIIc infection in sows and safeguarding the growth performance of newborn piglets.

In recent years, nanozymes—nanomaterials exhibiting enzymatic properties—have garnered significant attention in the biomedical field due to their high stability, low cost, and ease of modification (12). Among these, manganese dioxide (MnO_2_) nanozymes, with their unique catalase (CAT) and superoxide dismutase (SOD) mimetic activities, can catalyze the decomposition of excess H_2_O_2_ into H_2_O and O_2_ under physiological conditions, thereby effectively alleviating intracellular oxidative stress (13, 14). Furthermore, MnO_2_ nanozymes slowly release Mn^2+^ ions in acidic gastric fluid. Manganese not only serves as a cofactor for multiple antioxidant enzymes (e.g., Mn-SOD) but is also crucial for maintaining intestinal mucosal integrity (15).

Although MnO_2_ nanozymes show great potential in anti-inflammatory and antioxidant therapies, few studies have explored their application in alleviating growth retardation in piglets caused by PEDV GIIc infection. Therefore, this study was conducted in a commercial swine production setting to address the clinical problem of growth impairment in newborn piglets induced by PEDV GIIc infection. By orally administering different doses of MnO_2_ nanozymes to newborn piglets via gavage, we investigated their effects on growth performance, antioxidant capacity, and intestinal health. This study aims to provide a novel nutritional intervention strategy for mitigating the adverse effects of PEDV infection and to establish a theoretical basis for the clinical application of nanozymes in veterinary medicine.

## Materials and methods

### Animal experiment

To investigate whether MnO_2_ nanozymes can alleviate growth retardation in newborn piglets caused by PEDV GIIc infection in sows, a controlled trial was conducted. A total of 20 pregnant sows (Large White × Landrace, LY) were randomly divided into five experimental groups, with four replicates per group, each replicate comprising one sow. The 18-day trial commenced on the third day of farrowing, with MnO_2_ nanozymes solution administered via gastric gavage every other day. Detailed experimental design is presented in Table 1. The study was conducted at a commercial farm in Guizhou Province, with all procedures adhering to guidelines approved by the Animal Ethics Committee of Sichuan Agricultural University (Permit No.: SICAU 202412127). Daily diarrhea scores were recorded by designated personnel using the following criteria: 0 (normal); 1 (paste-like); 2 (semi-liquid); 3 (liquid) (16). For statistical analysis, diarrhea scores >1 were considered diarrhea. Fecal samples were collected at the end of the experiment. At 14 days of age, two piglets per litter with weights close to the litter average were selected. Blood was drawn from the superior vena cava into vacuum-tube blood collection tubes. Serum was separated by centrifugation at 3,500 rpm for 10 min at 4 C. Following euthanasia, samples including duodenum, jejunum, and ileum tissues were collected. All samples were obtained within 15 min post-slaughter and stored at appropriate temperatures. Lactating sows received the commercial farm's standard diet. The complete ingredient composition was not available from farm records; however, all groups were managed under the same basal feeding conditions, and piglets received sow milk as the basal diet. The experimental difference among treatment groups was oral MnO_2_ nanozyme supplementation. The MnO_2_ nanozyme dose gradient comprised 5, 10, and 20 mg/L to assess dose-related responses.

**Table 1:** Animal experimental design.

Group	Diet	Piglet number
NC	Breast milk	8
PC	Breast milk	8
5 mg/L	Breast milk + Oral 5 mg/L MnO_2_ nanozyme	8
10 mg/L	Breast milk + Oral 10 mg/L MnO_2_ nanozyme	8
20 mg/L	Breast milk + Oral 20 mg/L MnO_2_ nanozyme	8

### Synthesis of MnO_2_ nanozyme

The flower-like MnO_2_ nanozyme (MnO_2_ nanoflowers) used in this study was synthesized in our laboratory according to a previously reported method (17). Briefly, urea and MnSO4·H_2_O were dissolved in distilled water, followed by the slow addition of 0.2 M KMnO4. The mixture was kept at room temperature for 24 h. The precipitate was collected by centrifugation, washed alternately three times with distilled water and absolute ethanol, and dried at 60 C for 12 h.

### PEDV detection in rectal swabs

Before the experiment, rectal swabs were collected aseptically from each sow and placed into collection tubes containing sterile normal saline. Viral nucleic acids were extracted using the Viral DNA/RNA Extraction Kit (Xingchun [Changzhou] Biotechnology Co., Ltd., Changzhou, China) according to the manufacturer's instructions. PEDV RNA was detected using the real time PCR Porcine epidemic diarrhea virus (PEDV) Detection Kit (Pukang Tongchuang Biotechnology Co., Ltd., China) according to the manufacturer's instructions. The corresponding positive and negative controls were included in each assay. Results were recorded as cycle threshold (Ct) values, with lower Ct values indicating higher PEDV RNA levels.

### Intestinal morphology

Jejunal samples were first fixed in 4% paraformaldehyde solution for 8–24 h. Subsequently, tissues underwent dehydration, clearing, and embedding in paraffin to prepare 5-micron-thick sections. These sections were stained using hematoxylin-eosin staining to visualize gastrointestinal morphology. Observations were conducted using a Leica DM3000 microscope system (Leica Microsystems, Wetzlar, Germany). Measurement of villus height (from villus tip to crypt opening) and crypt depth (from crypt opening to base) was performed using ImageJ software.

### Antioxidant capacity

Total antioxidant capacity (T-AOC) and the activities of SOD, Mn-SOD, and CAT, as well as the concentrations of MDA and GSH, were determined using commercial assay kits from the Nanjing Jiancheng Institute of Bioengineering (Nanjing, China). All assays were performed according to the manufacturer's instructions.

### Serum biochemical testing

Serum biochemical parameters included total cholesterol (TC), triglycerides (TG), low-density lipoprotein (LDL), high-density lipoprotein (HDL), total protein (TP), albumin (ALB), globulin (GLB), aspartate aminotransferase (AST), and alanine aminotransferase (ALT), all of which were measured using a Hitachi 3100 fully automated biochemical analyzer.

### Total RNA extraction and real-time quantitative PCR (RT-PCR)

Total RNA was extracted from frozen duodenal, jejunal, and liver tissues using TRIzol reagent (Takara Bio Inc., Dalian, China), strictly following the manufacturer's protocol. RNA samples meeting quality standards were reverse transcribed using the PrimeScript™ Reverse Transcription Kit (Takara Bio Inc., Dalian, China) to synthesize cDNA. Relative RNA expression levels were quantified using the 2^∧^(–ΔΔCt) method with the housekeeping gene GAPDH for normalization. Specific primer pairs used for qPCR are detailed in Table 2.

**Table 2:** Primer sequences used in real-time PCR.

Primer ID	Forward primer sequence (5^′^-3^′^)	Reverse primer sequence (5^′^-3^′^)
GAPDH	ACTCACTCTTCCACTTTTGATGCT	TGTTGCTGTAGCCAAATTCA
ZnT10	GACAGGAAGGTCGAGAAGGG	GATGTTCAGGGCTTCGGACT
ZIP8	TGATGACTTTGGTCCTTCTCAGG	TCCATTAGGCTCTGTGACAGC
ZIP14	CGCACGAGCTAGGAGACTTT	CCAGACCCACGTAACAGCAG
MT1	GTGAATCCGCGTTGCTCTCTGCT	CTGTGGGGCAGGAGCAGTTGG
DMT1	AGGATCTAGGGCATGTGGTG	CCACAGTCCAGGAAGGACAT
ZO-1	TCAAGGTCTGCCGAGACAAC	ATCACAGTGTGGTAAGCGCA
Occludin	CAGGTGCACCCTCCAGATTG	ATGTCGTTGCTGGGTGCATA
Claudin-1	AACCGTGTGGGAACAACCAG	ACACATGAAAATGGCTTCCCT

### Gut microbiome analysis

Rectal content samples were sent to Novogene Co., Ltd. (Beijing, China) for 16S rRNA gene sequencing and analysis. Following DNA extraction, the V3–V4 region of the 16S rRNA gene was amplified using primers 341F (CCTAYGGGRBGCASCAG) and 806R (GGACTACNNGGGTATCTAAT). Raw sequence tags were processed using QIIME2. Sequences were compared with the SILVA database (https://www.arb-silva.de/) for taxonomic annotation, and chimeric sequences were removed to obtain the final valid dataset.

### Data analysis

Statistical analyses were performed using SPSS version 27, and bar charts were generated using GraphPad Prism version 9.5. Differences among groups were assessed by one-way analysis of variance (ANOVA) followed by Duncan's multiple range test. Data are presented as mean ± standard error of the mean (SEM), and *P*<*0.05* was considered statistically significant.

## Results

### Growth performance

Table 3 presents the rectal swab RT-qPCR Ct values obtained before the experiment. No significant differences were observed between sows in the PC group and those in the MnO_2_ nanozymes-administered groups. Reproductive performance was numerically better in the NC group than in the other four groups, but no significant differences were detected among groups. Animal trials were conducted to evaluate the effects of different concentrations of MnO_2_ nanozyme on the growth performance of newborn piglets; the results are shown in Table 4. Although piglets in the PC group had higher body weights at the start of the trial, by the end of the trial, the body weights of the NC group had significantly exceeded those of the PC group. Body weights in the MnO_2_ nanozyme gavage groups were not significantly different from those in the PC group and remained within the range observed in the NC group. Regarding average daily gain (ADG), piglets in the PC group had significantly lower ADG than those in the other four groups. No significant differences were observed among the MnO_2_ nanozyme groups at different concentrations, and ADG was slightly higher than that in the NC group. No statistically significant differences in diarrhea incidence or diarrhea index were detected among the treatment groups. These findings indicate that MnO_2_ nanozyme supplementation was associated with improved growth performance in piglets born to PEDV GIIc-infected sows; overall, the 5 mg/L concentration showed the most favorable overall response under the tested conditions.

**Table 3:** Rectal swab RT-qPCR Ct values and litter performance of sows.

Items	NC	PC	MnO_2_ (5 mg/L)	MnO_2_ (10 mg/L)	MnO_2_ (20 mg/L)	*P-*value
Rectal swab	0.00 ± 0.00^b^	34.26 ± 1.63^a^	34.03 ± 0.34^a^	33.45 ± 0.75^a^	35.17 ± 0.29^a^	< 0.001
Healthy piglets, head	12.00 ± 0.82	11.25 ± 0.85	9.00 ± 0.91	10.25 ± 0.85	11.50 ± 1.19	0.220
Weak piglets, head	1.50 ± 0.65	0.50 ± 0.50	0.48 ± 0.50	0.50 ± 0.25	0.25 ± 0.70	0.459
Stillbirths, head	0.50 ± 0.50	0.25 ± 0.25	0.50 ± 0.29	0.00 ± 0.00	0.50 ± 0.29	0.721
Total litter size, head	14.00 ± 1.08	12.00 ± 1.22	10.25 ± 1.31	10.75 ± 0.48	12.25 ± 0.85	0.146

Data are presented as mean ± SEM (*n* = 4 litters per group). Within a row, values with different superscript letters differ significantly (*P* < 0.05).

**Table 4:** Effect of different concentrations of MnO_2_ nanozyme on growth performance of newborn piglets.

Items	NC	PC	MnO_2_ (5 mg/L)	MnO_2_ (10 mg/L)	MnO_2_ (20 mg/L)	*P-*value
3 d weight, kg	1.33 ± 0.24^b^	1.64 ± 0.08^ab^	2.18 ± 0.18^a^	2.17 ± 0.22^a^	1.73 ± 0.11^ab^	0.016
14 d weight, kg	4.12 ± 0.53^ab^	3.25 ± 0.48^b^	5.18 ± 0.31^a^	4.96 ± 0.29^a^	4.58 ± 0.19^ab^	0.019
21 d weight, kg	5.38 ± 0.59^ab^	3.58 ± 0.72^b^	6.65 ± 0.37^a^	6.39 ± 0.43^a^	6.08 ± 0.27^a^	0.004
3-21 d ADG, g/d	225.35 ± 21.16^a^	108.12 ± 40.08^b^	248.44 ± 10.54^a^	234.63 ± 25.34^a^	241.74 ± 11.14^a^	0.005
Diarrhea incidence, %	0.53 ± 0.53	0.36 ± 0.36	0.13 ± 0.13	0.27 ± 0.15	0.34 ± 0.20	0.929
Diarrhea index	0.0132 ± 0.013	0.0010 ± 0.001	0.0040 ± 0.004	0.0063 ± 0.007	0.0048 ± 0.003	0.744
Mortality, %	2.56	4.88	2.50	0	0	0.292

Data are presented as mean ± SEM (*n* = 8 piglets per group). Within a row, values with different superscript letters differ significantly (*P* < 0.05).

### Intestinal morphology

Figure 1 illustrates the morphological characteristics of the jejunum in 14-day-old piglets. Compared with the NC group, the PC group showed impaired intestinal morphology, including markedly shortened intestinal villi (Figures 1A–E, F), no significant difference in crypt depth (Figures 1A–E, G), and a reduced villus height-to-crypt depth ratio (Figures 1A–E, H). Following oral administration of MnO_2_ nanozymes, villus height and the villus height-to-crypt depth ratio were restored to levels comparable to those in the NC group (Figures 1A–F, H). However, crypt depth in the 20 mg/L gavage group was significantly reduced compared with both the NC and PC groups (Figures 1A–E, G). In addition, jejunal epithelial cells were more regularly arranged in the MnO_2_ nanozymes-supplemented groups (Figures 1A–E). No significant differences were observed in jejunal *ZO-1, Occludin*, or *Claudin-1* gene expression among groups. Overall, the 5 mg/L MnO_2_ gavage group showed intestinal barrier-related gene expression closest to that of the NC group (Figures 1I–K).

**Figure 1:**
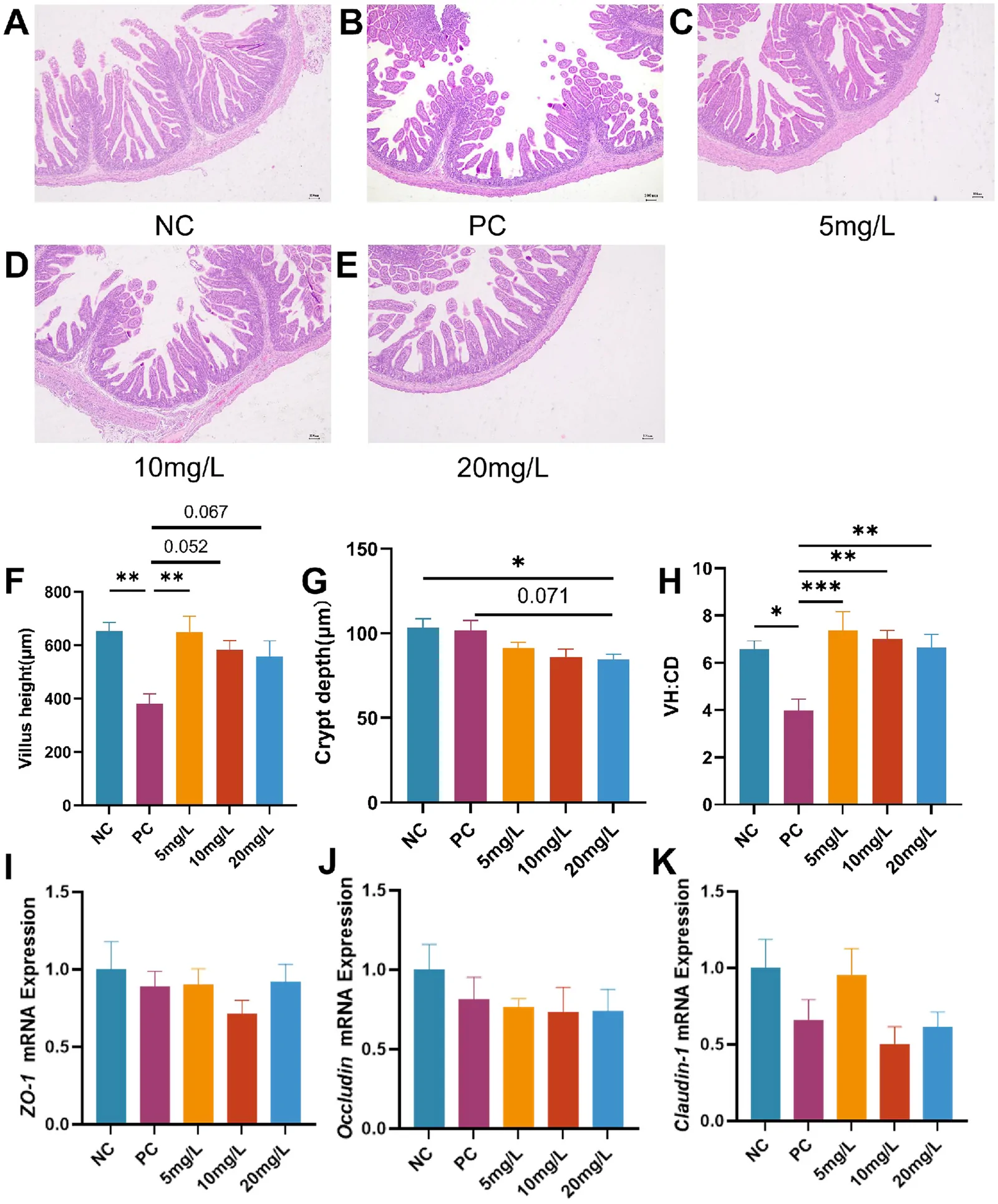
**(A–E)** Morphology of the jejunum in neonatal piglets; **(F)** villus height of the jejunum in neonatal piglets; **(G)** crypt depth of the jejunum in neonatal piglets; **(H)** villus height-to-crypt depth ratio of the jejunum in neonatal piglets; **(I)** ZO-1 mRNA expression in the jejunum of neonatal piglets; **(J)** Occludin mRNA expression in the jejunum of neonatal piglets; **(K)** Claudin-1 mRNA expression in the jejunum of neonatal piglets. *P < 0.05, **P < 0.01, and ***P < 0.001. Data are presented as mean ± SEM (*n* = 8 piglets per group).

### Antioxidant capacity

Figure 2 presents serum antioxidant and oxidative stress-related parameters in piglets. Compared with the NC group, the PC group showed significantly decreased serum CAT and T-SOD activities (Figures 2B, C). No significant differences were observed in T-AOC, GSH, MDA, or Mn-SOD levels (Figures 2A, D–F). Following MnO_2_ nanozymes supplementation, CAT and T-SOD activities improved, with an apparent dose-related trend (Figures 2B, C). In the high-dose (20 mg/L) group, serum T-AOC and Mn-SOD activity also showed an upward trend (Figures 2A, F). No significant differences in serum MDA levels were detected among groups. Overall, serum antioxidant status in the 20 mg/L MnO_2_ nanozymes group was closest to that of the NC group.

**Figure 2:**
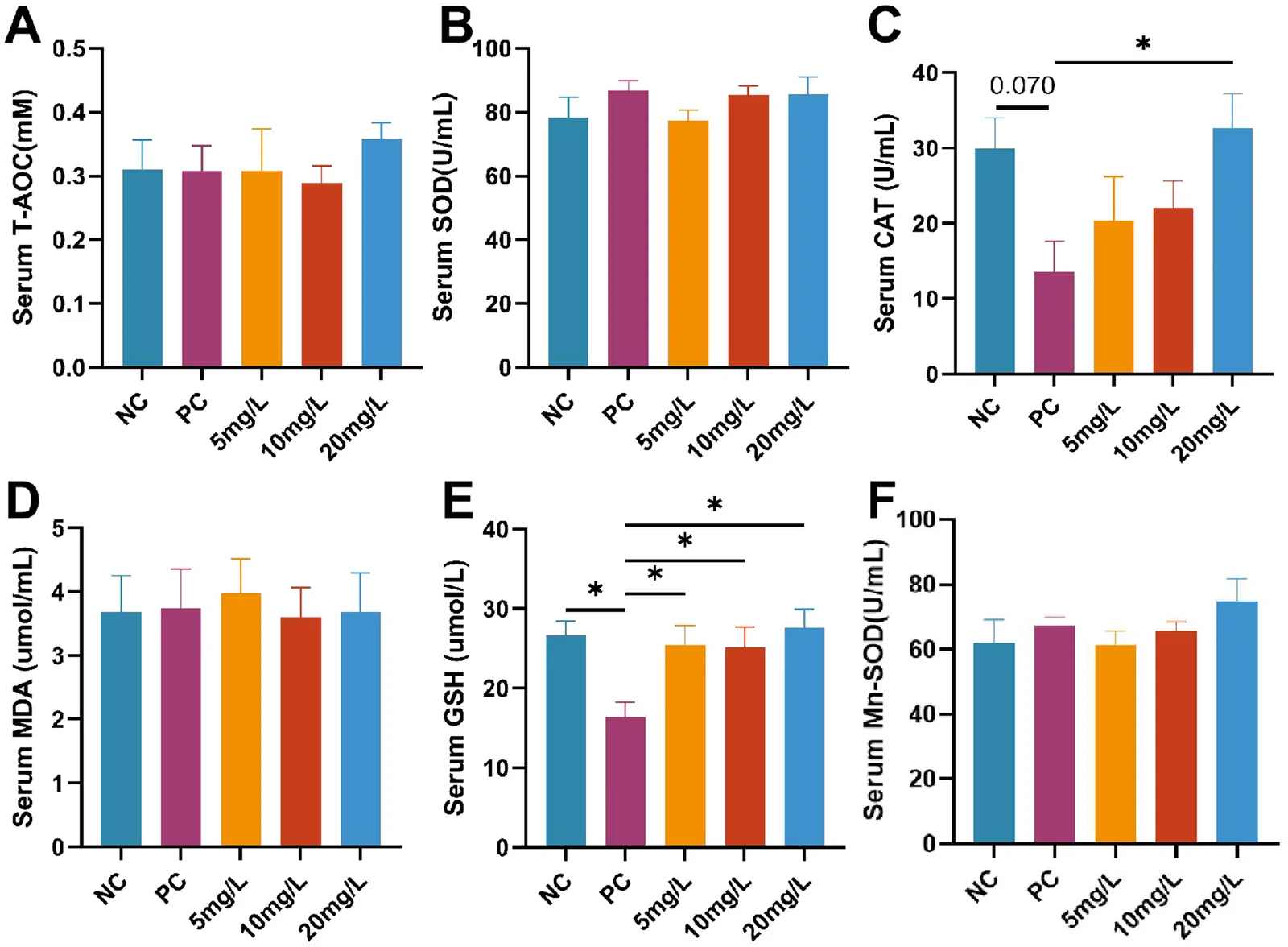
**(A)** Total antioxidant capacity in serum; **(B)** total superoxide dismutase activity in serum; **(C)** catalase activity in serum; **(D)** malondialdehyde content in serum; **(E)** glutathione content in serum; **(F)** manganese superoxide dismutase activity in serum. *P < 0.05 and **P < 0.01. Data are presented as mean ± SEM (*n* = 8 piglets per group).

Figure 3 presents the changes in antioxidant and lipid peroxidation-related parameters in the jejunum and liver tissues of piglets. Compared with the NC group, the local tissue antioxidant defense system in the PC group was impaired to a certain extent, as evidenced by decreased levels of jejunal CAT, jejunal GSH, and hepatic GSH (Figures 3A, B, E), whereas no significant changes were observed in hepatic CAT, jejunal MDA, or hepatic MDA levels (Figures 3C, D, F). Notably, supplementation with MnO_2_ nanozymes significantly alleviated these impaired antioxidant parameters in piglet tissues. Specifically, nanozyme administration effectively promoted a gradual recovery of jejunal CAT and GSH levels, with the recovery of jejunal GSH exhibiting a clear dose-dependent relationship (Figures 3A, B). Concurrently, hepatic GSH levels were also significantly increased following nanozyme intervention (Figures 3E). Regarding tissue lipid peroxidation, MDA levels in both the jejunum and liver remained essentially stable across all treatment groups, with no significant fluctuations observed (Figures 3C, F). Overall, the 20 mg/L MnO_2_ nanozymes group demonstrated the most pronounced effect on restoring comprehensive antioxidant function in the jejunum; however, even the 5 mg/L MnO_2_ nanozymes group was sufficient to restore the antioxidant status of piglet tissues to near-normal levels.

**Figure 3:**
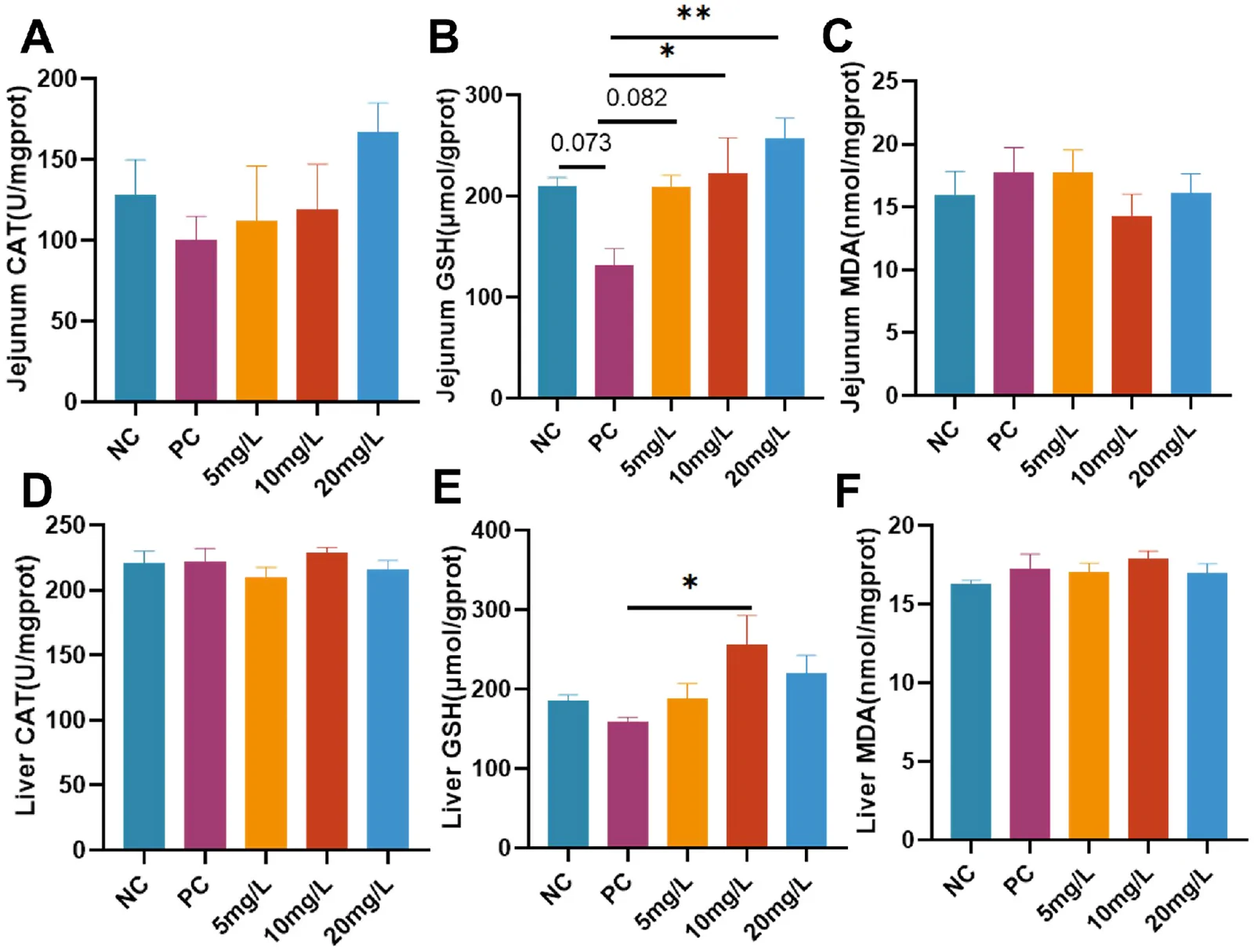
**(A)** Catalase activity in jejunum; **(B)** glutathione content in jejunum; **(C)** malondialdehyde content in jejunum; **(D)** catalase activity in liver; **(E)** glutathione content in liver; **(F)** malondialdehyde content in liver. *P < 0.05 and **P < 0.01. Data are presented as mean ± SEM (*n* = 8 piglets per group).

### Serum biochemistry

Serum protein, lipid, and transaminase parameters were measured across all groups to assess protein synthesis, lipid metabolism, and liver-related biochemical status. No significant differences were observed in total protein or globulin levels among groups, and these parameters remained generally stable (Figures 4A, C). Albumin levels were slightly lower in the PC group than in the NC group, whereas the intervention groups showed varying degrees of recovery (Figure 4B). No significant intergroup differences were detected in TC or LDL; HDL showed slight fluctuations, suggesting that lipid metabolism was largely unaltered (Figures 4D–F). AST and ALT levels were relatively higher in the NC group and the 20 mg/L nanozyme-treated group than in the other groups (Figures 4G, H). The AST/ALT ratio did not differ significantly among groups (Figure 4I).

**Figure 4:**
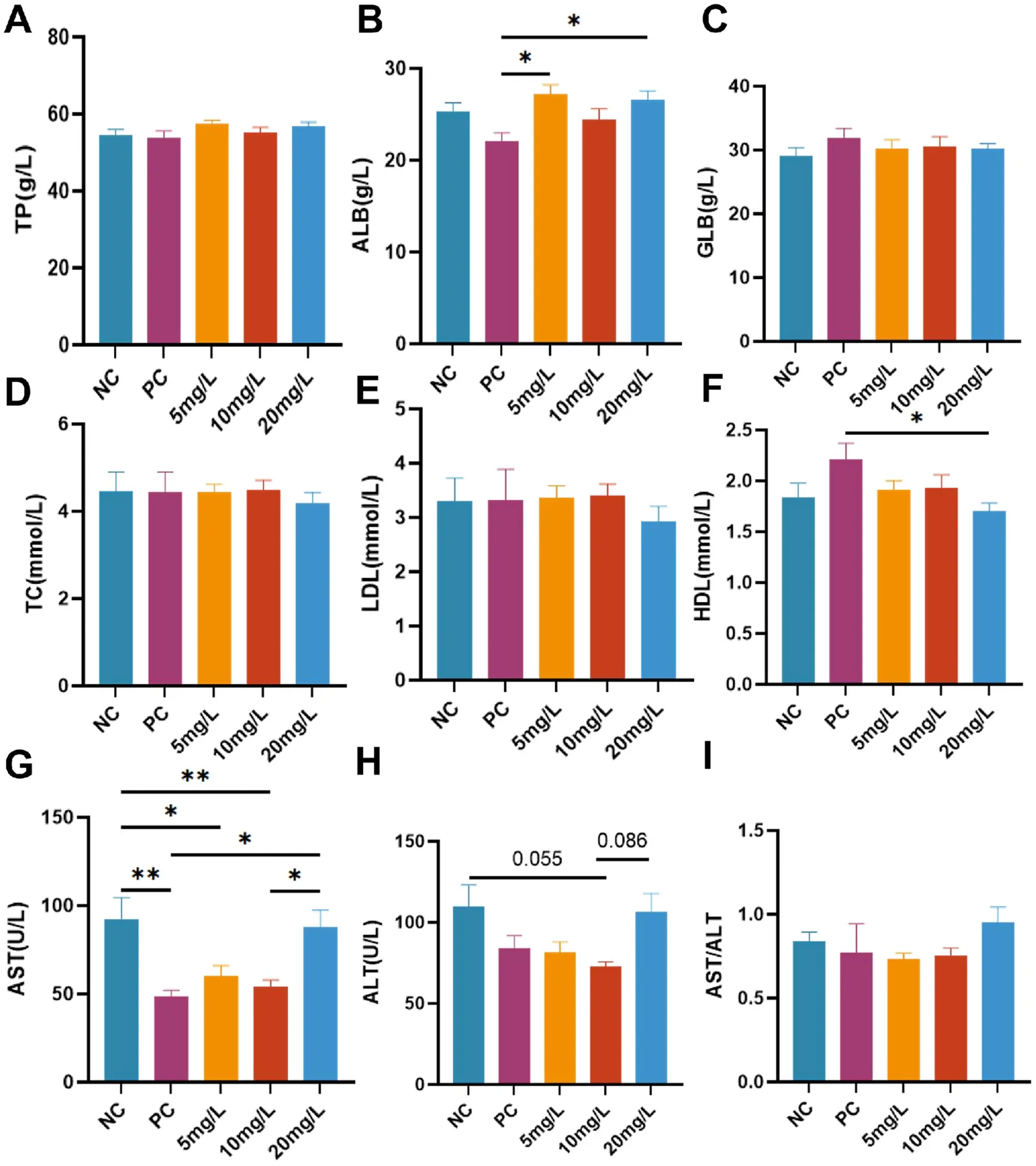
**(A–I)** Serum levels of total protein (TP), albumin (ALB), globulin (GLB), total cholesterol (TC), low-density lipoprotein (LDL), high-density lipoprotein (HDL), aspartate aminotransferase (AST), alanine aminotransferase (ALT), and AST/ALT. *P < 0.05 and **P < 0.01. Data are presented as mean ± SEM (*n* = 8 piglets per group).

### Manganese metabolism

Manganese is an essential trace element involved in multiple physiological processes that support piglet growth and development. Dietary manganese deficiency has been reported to increase intestinal permeability by disrupting intestinal tight junctions (18). To investigate the potential effects of MnO_2_ nanozyme supplementation on manganese-related transport, we assessed mRNA expression of manganese transporter genes in the duodenum and liver. Figure 5 shows changes in manganese-related transporter gene expression and duodenal manganese content. Compared with the NC group, the PC group showed lower duodenal manganese content (Figure 5F) and reduced mRNA expression of *ZIP8, ZIP14, DMT1, ZnT10*, and *MT1* (Figures 5A–E). Following MnO_2_ nanozymes supplementation, *DMT1* expression and duodenal manganese content increased, with an apparent dose-related pattern (Figures 5C, F). The 20 mg/L group showed the largest increase in duodenal manganese content and *DMT1* expression among the supplemented groups.

**Figure 5:**
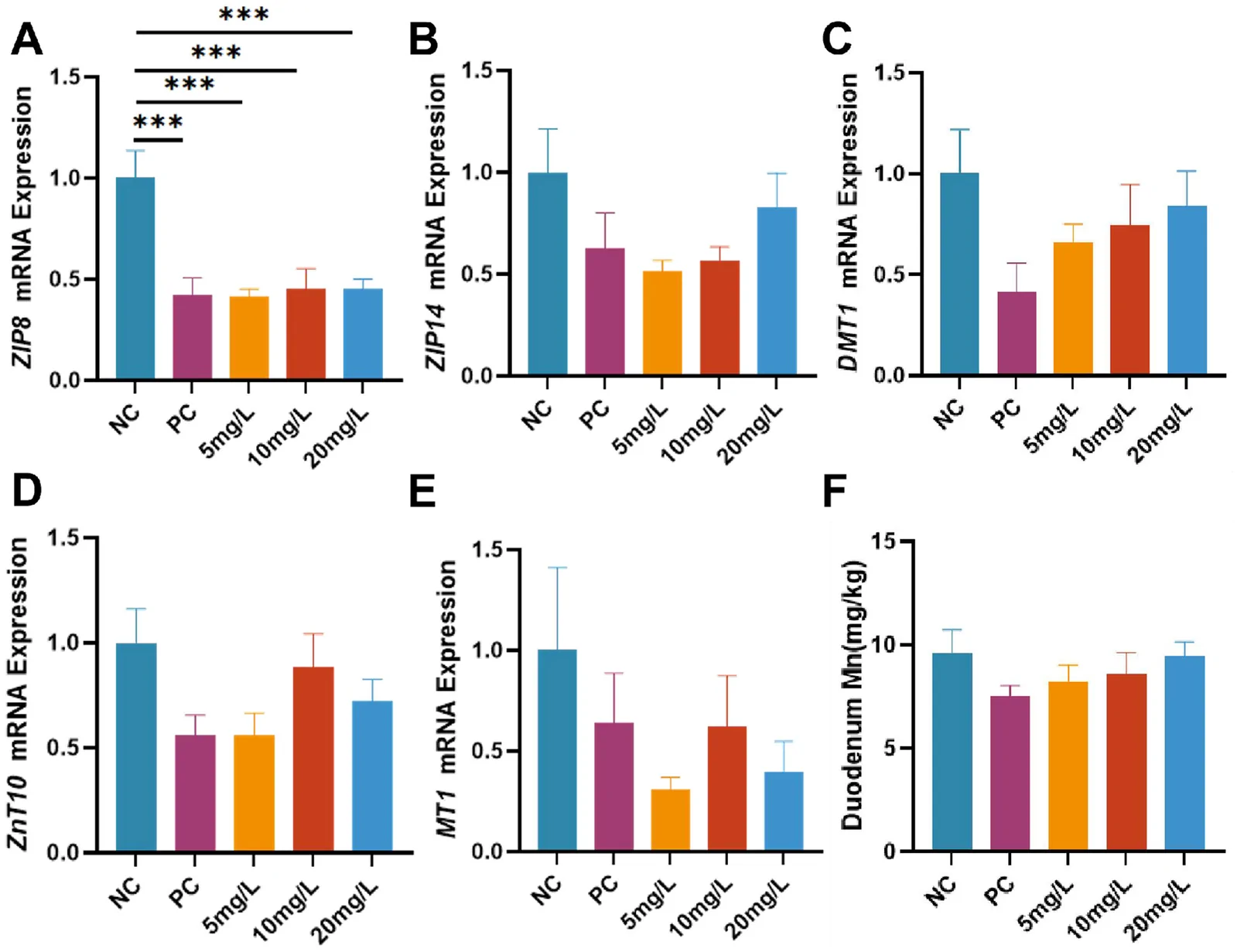
**(A)** ZIP8 mRNA expression in the duodenum of neonatal piglets; **(B)** ZIP14 mRNA expression in the duodenum of neonatal piglets; **(C)** DMT1 mRNA expression in the duodenum of neonatal piglets; **(D)** ZnT10 mRNA expression in the duodenum of neonatal piglets; **(E)** MT1 mRNA expression in the duodenum of neonatal piglets; **(F)** duodenal manganese content in neonatal piglets. *P < 0.05, **P < 0.01, and ***P < 0.001. Data are presented as mean ± SEM (*n* = 8 piglets per group).

Figure 6 shows changes in manganese-related transporter gene expression and tissue manganese content in piglet livers. Compared with the NC group, the PC group showed increased hepatic manganese content (Figure 6F). The mRNA expression levels of *ZIP8, ZIP14*, and *DMT1* were increased (Figures 6A–C), whereas *ZnT10* and *MT1* expression was decreased (Figures 6D, E). Following MnO_2_ nanozyme supplementation, hepatic manganese content and the expression of several manganese-related transporters changed relative to the PC group. In particular, hepatic manganese content was lower in the supplemented groups than in the PC group (Figure 6F). The 20 mg/L group showed the most pronounced overall changes in hepatic manganese-related indices; however, these measurements do not directly establish manganese flux or whole-body manganese homeostasis.

**Figure 6:**
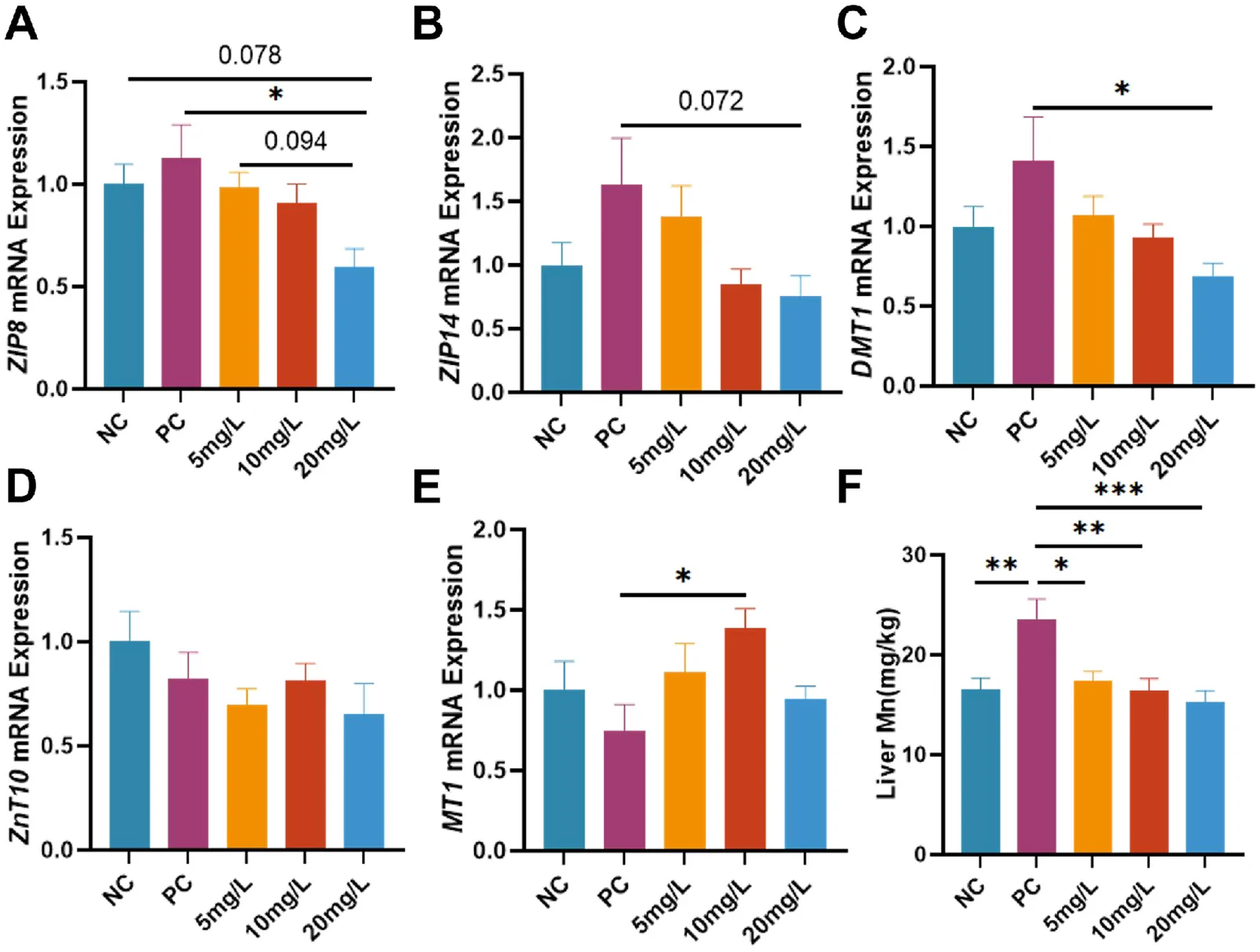
**(A)** ZIP8 mRNA expression in the liver of neonatal piglets; **(B)** ZIP14 mRNA expression in the liver of neonatal piglets; **(C)** DMT1 mRNA expression in the liver of neonatal piglets; **(D)** ZnT10 mRNA expression in the liver of neonatal piglets; **(E)** MT1 mRNA expression in the liver of neonatal piglets; **(F)** hepatic manganese content in neonatal piglets. *P < 0.05, **P < 0.01, and ***P < 0.001. Data are presented as mean ± SEM (*n* = 8 piglets per group).

### Gut microbiota

The composition of the rectal fecal microbiota was analyzed via 16S rRNA sequencing. A petal diagram analysis (Figure 7A) revealed substantial overlap in ASV composition among groups. Across all five groups, a total of 3,399 amplicon sequence variants (ASVs) were identified, with 291 ASVs shared among all groups (core microbiome). Each group also possessed unique ASVs: 521 in the NC group, 546 in the PC group, 626 in the 5 mg/L group, 649 in the 10 mg/L group, and 572 in the 20 mg/L group. α-diversity analysis indicated a trend toward increased gut microbial abundance following MnO_2_ nanozymes administration at concentrations below 10 mg/L (Figure 7B). Principal coordinate analysis (PCoA) based on unweighted UniFrac distances revealed differences in gut microbiota structure among the five groups. As shown in Figure 7C, samples from each group exhibited mixed distribution without distinct clustering, indicating highly similar community structures. At the phylum level, the sum of relative abundances for Firmicutes and Bacteroidetes showed no significant differences between groups (Figure 7D). At the genus level, the relative abundance of *Prevotella* in the 5 mg/L and 10 mg/L groups was comparable to that in the NC group, while the relative abundance of *Alloprevotella* showed a significant increase (Figure 7E). LEfSe analysis identified characteristic signature microbial communities (LDA score > 3.0) across the five sample groups: T1 (NC group) was enriched with *Barnesiella* and related taxa; T2 (PC group) was dominated by *Parabacteroides, Tannerellaceae*, and other differential bacteria; T3 (5 mg/L group) was enriched with *Prevotellaceae*-related taxa, which had the highest LDA score among all species; T5 (20 mg/L group) was primarily enriched with *Clostridiales* and *Clostridium*-related taxa. The microbial composition characteristics of each group were distinctly different, but the 10 mg/L group showed no significant differential bacterial communities (Figure 7F).

**Figure 7:**
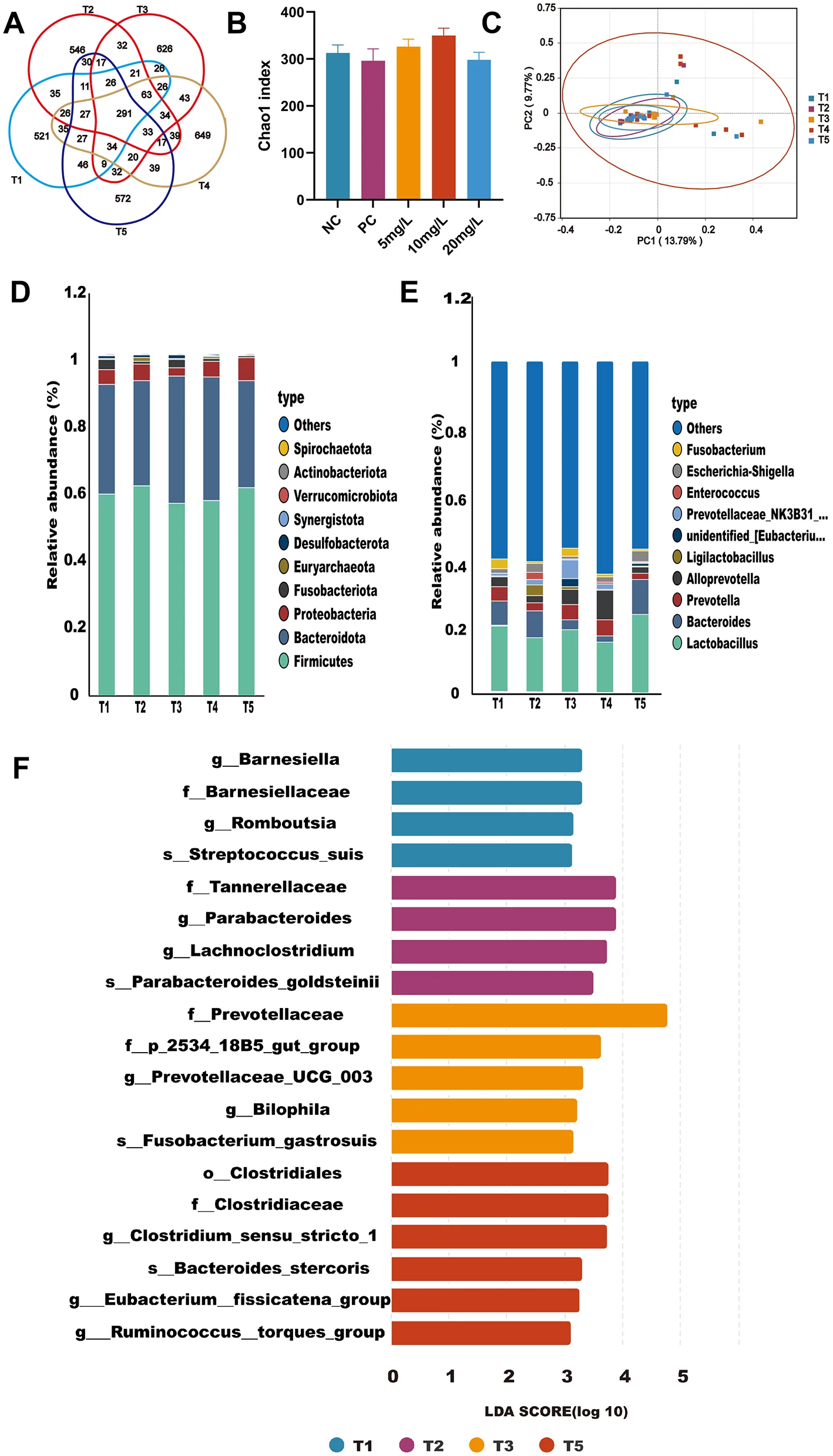
**(A)** Venn analysis of amplicon sequence variants (ASVs) of the rectal bacterial community; **(B)** alpha diversity indexes; **(C)** principal coordinate analysis (PCoA) of the rectal bacterial community; **(D)** bar plots at the phylum level; **(E)** bar plots at the genus level; **(F)** histogram of LDA scores for differential microbial biomarkers identified by LEfSe analysis. T1, NC group; T2, PC group; T3, 5 mg/L group; T4, 10 mg/L group; T5, 20 mg/L group. Data are based on biological replicates (n = 8 piglets per group).

Spearman correlation analysis based on individual paired data was conducted between the selected rectal fecal bacterial genera and physiological parameters. *Parabacteroides* abundance showed a negative association with the villus height-to-crypt depth ratio (ρ = −0.44, unadjusted *P* = 0.005); however, this association did not remain significant after false discovery rate correction (*q* = 0.078). No other associations between the selected genera and physiological parameters reached statistical significance after FDR correction (Figure 8).

**Figure 8:**
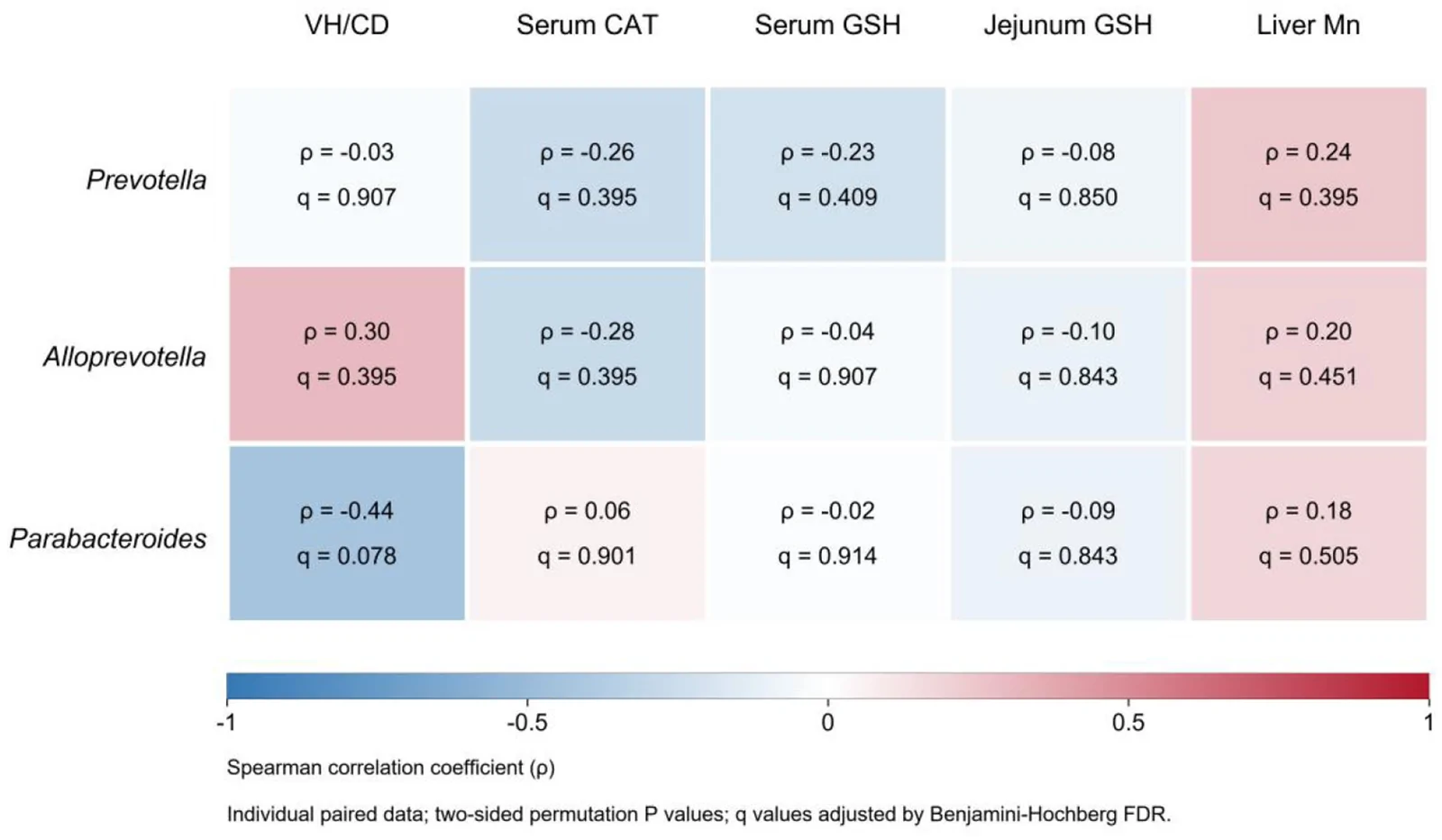
Spearman correlation analysis between selected rectal fecal bacterial genera and physiological parameters in piglets. Cells show Spearman correlation coefficients (ρ) and FDR-adjusted q values. Correlations were calculated using individual paired data.

## Discussion

This study investigated whether oral MnO_2_ nanozymes administration could alleviate PEDV GIIc-associated growth retardation in piglets born to infected sows. The findings indicate that nanozyme administration was associated with improved growth performance, intestinal morphology, antioxidant status, manganese-related indices, and selected rectal fecal microbial features.

PEDV infection can cause intestinal epithelial damage, villous atrophy, barrier dysfunction, and impaired nutrient absorption, thereby contributing to growth retardation in neonatal piglets (1, 19, 20). In the present study, the lower ADG in the PC group was consistent with this PEDV-associated growth-suppression phenotype (21). MnO_2_ nanozymes administration increased final body weight and ADG relative to the PC group. Baseline body weight differed among groups on day 3 and may have influenced subsequent growth; nevertheless, the 20 mg/L group, whose initial body weight was comparable to that of the PC group, also showed higher final body weight and ADG. This supports a beneficial association between MnO_2_ nanozymes administration and growth performance, while the influence of baseline body weight should be considered. Although PEDV commonly causes diarrhea in neonatal piglets, diarrhea incidence and diarrhea index did not differ significantly among groups. The sows were in the later stage of infection and had been vaccinated, which may have reduced clinical severity in their piglets through residual maternal protection. However, the absence of a diarrhea difference does not exclude PEDV GIIc-associated intestinal injury or growth-related effects.

Morphological observations showed that nanozyme administration increased jejunal villus height and improved the villus height-to-crypt depth ratio. This finding is consistent with reports of MnO_2_ nanozymes-mediated improvement of intestinal mucosal injury in inflammatory models (22, 23). The lower crypt depth observed after MnO_2_ nanozymes administration may reflect reduced injury and compensatory mucosal turnover, but, the specific mechanisms require further study.

Oxidative stress is an important contributor to PEDV-related intestinal barrier injury, and neonatal piglets have an underdeveloped endogenous antioxidant system (24, 25). MnO_2_ nanozymes have catalase-like activity and can participate in redox regulation (17). In this study, the increased CAT activity and serum GSH in the 20 mg/L group, together with improvements in jejunal antioxidant indices, are consistent with improved local and systemic antioxidant capacity. These measurements do not directly demonstrate ROS scavenging *in vivo*, but support the proposed antioxidant role of MnO_2_ nanozymes (26, 27).

Analysis of serum biochemical parameters indicates that this nanozyme exhibits good *in vivo* biocompatibility and metabolic safety within a concentration range of 5–20 mg/L (28). During the experiment, lipid profiles (TC, LDL, HDL) and baseline protein levels (total protein, globulin) remained stable in all groups, with no metabolic disturbances observed. Furthermore, nanoenzyme intervention effectively restored impaired albumin levels, suggesting that it helps maintain or even improve hepatic protein synthesis function (29). Furthermore, although liver transaminase (AST, ALT) levels in the high-dose group (20 mg/L) exhibited fluctuations comparable to those in the control group, the AST/ALT ratio remained constant across all groups, clearly ruling out the risk of organic liver damage and hepatic parenchymal necrosis (30), further confirming the high safety profile of this nanozyme for *in vivo* application.

In this study, PEDV challenge reduced duodenal Mn content and manganese transporter expression, while increasing hepatic Mn accumulation and the expression of uptake-related transporters such as *ZIP8, ZIP14*, and *DMT1*. This opposite pattern suggests a nutrition-immunity-like disturbance of manganese distribution along the intestine-liver axis (31). In the duodenum, the PC group exhibited impaired manganese absorption, whereas oral administration of MnO_2_ nanozymes (particularly at 10–20 mg/L) effectively restored the gene expression of key transmembrane transporters such as *DMT1* and *ZIP14*, improved baseline intestinal absorption function, and gradually returned tissue manganese levels to normal (32). Conversely, in the liver—a key metabolic organ—the PC group exhibited significant pathological manganese overload and abnormally high expression of uptake proteins (*ZIP8, ZIP14, DMT1*); In contrast, the intervention groups effectively reversed this phenomenon. Not only did they downregulate the overall uptake pathways and block the abnormal influx of manganese to significantly reduce hepatic manganese accumulation, but the 5–10 mg/L dose group also significantly upregulated the expression of metallothionein *MT1*, effectively enhancing the liver's endogenous metal detoxification capacity (33).

The 5 mg/L and 10 mg/L groups showed changes in the relative abundance of *Prevotella*- and *Alloprevotella*-related taxa. *Prevotella*-associated short-chain fatty acid metabolism has been linked to epithelial energy supply, inhibition of pathogen growth through reduced pH, and intestinal barrier function (34–36). Although overall microbial community structures were similar across groups, the increase in key functional genera indicates that MnO_2_ nanozymes create favorable “hypoxic/low-oxidative” niches for the colonization of obligate anaerobic beneficial bacteria by improving the intestinal redox microenvironment (37). In the association analysis, the association between VH:CD and microorganisms was relatively clear, Parabacteroides abundance showed a nominal negative association with the villus height-to-crypt depth ratio, but this association was not significant after false discovery rate correction.

In conclusion, MnO_2_ nanozymes administration was associated with alleviation of PEDV GIIc-associated growth retardation in piglets born to infected sows, accompanied by changes in intestinal morphology, antioxidant capacity, manganese-related indices, and rectal fecal microbiota. Among the tested treatments, 5 mg/L showed a favorable overall response at the lowest administered concentration. Further controlled studies are needed to confirm the mechanisms, optimize the dose, and clarify microbiota-host relationships.

## Data Availability

The data presented in the study are deposited in the NCBI Sequence Read Archive (SRA) repository, accession number PRJNA1520866.
